# Why are medical students so motivated to learn ultrasound skills? A qualitative study

**DOI:** 10.1186/s12909-024-05420-3

**Published:** 2024-04-26

**Authors:** Anina Pless, Roman Hari, Michael Harris

**Affiliations:** 1 Institute of Primary Health Care (BIHAM), University of Bern, Mittelstrasse 43, 3012 Bern, Switzerland; 2 College of Medicine & Health, University of Exeter Medical School, Exeter, UK

**Keywords:** Motivation, Qualitative research, Ultrasound, Medical education

## Abstract

**Background:**

The introduction of ultrasound (US) courses into medical undergraduate courses is usually met with a particularly high level of student motivation. The reasons for this are unclear. The aim of this study was to investigate the factors that contribute to undergraduate medical students’ motivation to learn US skills. Understanding what motivates students to learn US will inform the efforts of faculty to foster students’ motivation to learn.

**Methods:**

We carried out in-depth semi-structured one-to-one interviews with medical students participating in an optional US course at two Swiss universities. The interview guide consisted of 10 main questions. The content was informed by experts in the field of medical education and US, as well as by a literature review of motivation theories for learning, in particular by self-determination theory (SDT). SDT was used to guide the development of the interview guide and to reflect on the resulting themes in the discussion section. The interview guide was piloted with two medical students. The interviews lasted an average of 45 min and were audio recorded and transcribed. Thematic analysis was used to analyse the data.

**Results:**

Fourteen undergraduate medical students in their preclinical (year 3) and clinical studies (years 4 and 5) elaborated on a wide range of reasons for their high motivation to learn US. They were motivated for US training because of the positive nimbus of the US modality, emphasising the advantages of visualisation. Students acknowledged the potential professional benefits of learning US and described it as a fun, exciting group activity.

**Conclusions:**

The four themes we found in our analysis can all be related to the three universal needs described in SDT. The strong focus on the visual aspect and the positive nimbus of the modality goes beyond that and reflects the visuo-centric Zeitgeist, which claims the superiority of visual information over other data. Educators should be aware that motivation to learn is affected by the Zeitgeist and ensuing preconceptions, such as the perception of the positive nimbus surrounding a topic. Other key elements that can be implemented to motivate students are just-in-time feedback, enabling group experiences and creating awareness of the clinical relevance of learning content.

**Supplementary Information:**

The online version contains supplementary material available at 10.1186/s12909-024-05420-3.

## Background

The introduction of mandatory ultrasound (US) courses at our University was greeted with a standing ovation. This is not the usual reaction to the implementation of new courses and is worth exploring. We hope to understand factors that contribute to students’ motivation for learning to foster such factors when teaching.

High levels of motivation correlate with better grades throughout medical studies [1, 2]. Kusurkar et al. examined “the effect of quality of motivation on performance” and found that motivation stemming from personal interest positively affects academic performance [3]. There is a positive correlation between motivation from personal interest and the intention to continue ones studies [1]. Motivation seems also to be correlated with learners’ well-being [4].

**Fig. 1:**
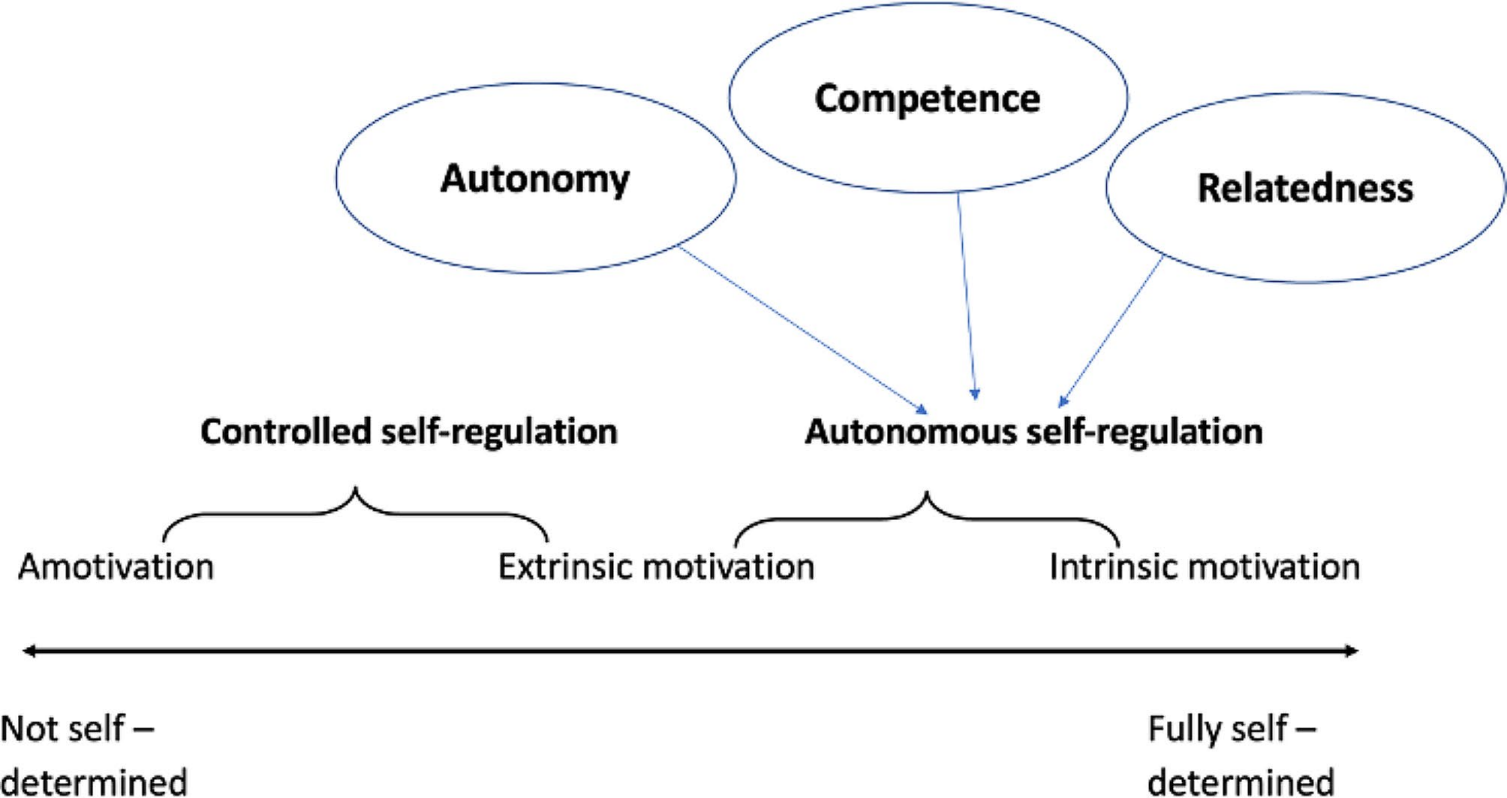
The Self-Determination Continuum, adapted from Ryan and Deci, 2000, Cook and Artino, 2016 and ten Cate et al., 2011

Several theories try to explain why students are motivated to learn. According to Self-determination theory (SDT), developed by Deci and Ryan, motivation varies not only in quantity but also in quality [5]. A central idea of the theory is that there is a distinction between autonomous motivation and controlled motivation. Autonomous motivation can be defined as engaging in an activity because one finds it interesting: doing something of one’s own free will. Controlled motivation is the norm, especially after early childhood, when social demands and roles require individuals to take responsibility for non-intrinsically interesting tasks. Students’ motivation to learn ultrasound, as perceived in our experience and as described in the literature, would mainly be classified as “autonomous” motivation in SDT, as they are engaging in an activity that they find interesting [6–8]. Three universal psychological needs add to the autonomous motivation: autonomy (being free to choose whatever one considers useful to do), competence (the desire to master a task and to feel effective) and relatedness (the feeling of wanting to connect with others and have a sense of belonging) [8, 9]. Figure 1 depicts states of motivated behaviour and its relation to autonomy, competence and relatedness. At one end of the continuum is amotivation, the state of lacking an intention to act. When amotivated, people either do not act at all or they act without purpose. Amotivation results from not appreciating an activity, not feeling competent to do it, or not expecting it to achieve a desired outcome. Intrinsic motivation is placed at the other end of the continuum, emphasising that it is the essential form of self-determined activity, based solely on an internal desire to act. Extrinsically motivated behaviours cover the states between amotivation and intrinsic motivation, varying in the extent to which their regulation is autonomous [7]. 

Our observation of students’ reception of the implementation of US teaching is in line with recent evidence [10]. Evaluations of US training programmes report positive feedback from over 95% of students, approval rates rarely seen in other fields of medical education [11, 12]. In a USA study, 97% of students agreed that it was important for them to learn basic US skills during medical school [13]. A German questionnaire study of medical students found a similar number (98.2%) reporting a high or very high interest in curricular US in medical education, with the large majority (94.4%) of respondents agreeing or strongly agreeing that US education should be a mandatory part of their curriculum [14]. In another study, a large majority of teachers (97%) felt that the students were interested in the course [15]. Another development that underlines the attractiveness of US education for students is the introduction of extracurricular, student-led peer-tutoring initiatives [16].

The scarce evidence on why students are motivated to learn US skills, suggests that they believe that US education helps them to be prepared for clinical practice before and after graduation [14, 17]. They also describe educational benefits beyond the mastering of US skills, for example learning physical examination skills or improving their understanding of anatomy and physiology [11, 13, 14, 18]. In a recent study, Wang et al. explored why pre-clinical medical students wanted to learn US by applying Existence, Relatedness and Growth Theory, a model with three categories often used for analysing employee work performance. Students gave reasons related to all three categories, foremost existence needs (e.g. future work requirement) and growth needs (e.g. improving diagnostic skills), with relatedness needs being mentioned less often [19]. Given SDT’s three basic psychological needs of autonomy, competence and relatedness, the spectrum of reasons is likely to be broader than this.

The aim of this study was to investigate the factors that contribute to undergraduate medical students’ motivation to learn US skills, using SDT as a conceptual framework.

## Methods

### Study design

This was a qualitative study following a constructivist paradigm. We used semi-structured one-to-one interviews to allow the exploration of sensitive topics and motivators. We used thematic analysis to analyse the data [20, 21]. We adhered to the Consolidated Criteria for Reporting Qualitative Research Checklist (COREQ) in the reporting of this study (for full COREQ checklist see supplementary Appendix 1) [22].

### Study setting and participants

We interviewed 14 medical students from two different Swiss medical schools (University of Bern, University of Zurich) between 2019 and 2022. Both Universities had developed identical, optional medical student courses in abdominal US education that used volunteer peer-tutors. The blended learning programme was available to all Swiss medical students. The ultrasound course curriculum for the students consisted of five modules, with five hours of e-learning and 16 hours of peer-led tutoring. After completing a summative practical assessment, participants could obtain an internationally recognised course certificate in abdominal ultrasound [23]. Approximately 20% of medical students participated in these courses.

### Recruitment and sampling method

We used purposive sampling. We contacted all the students that were enrolled in a voluntary, extracurricular US course (*n* = 95). Potential participants were invited by an email forwarded to them by the course organisers and were also approached directly at US courses. While we had aimed to stratify participants with regards to gender, University site and age, recruitment was difficult during the COVID-19 pandemic, so we interviewed all the students willing to participate. All the students who agreed to take part had already participated in at least two tutored sessions and gave written, informed consent. After initial contact had been made, none of the participants declined to take part in the study or dropped out of it.

### Ethical considerations

The study was submitted to the regional ethics committee which stated that the study does not fall under the Swiss Human Research Act (BASEC-Nr. Req-2018-00059).

All participants gave written informed consent. To ensure participant anonymity, each participant was identified only by a code after interview transcription.

### Development of the interview guide

The semi-structured interview guide was informed by a literature review of motivation theories for learning, in particular by SDT, and discussions with faculty who are involved in US education, including medical educators, clinicians and US experts. Based on this information the research team discussed how to phrase questions that would be broad enough to encompass a variety of factors which can contribute to motivation according to SDT [7, 9]. The interview guide consisted of 10 main questions which focussed on participants’ reasons to learn US and the perceived benefits (see Appendix 2). Each main question had probes to get more in-depth information. The interview guide was piloted with two students, and changes were made where necessary.

### Data collection

All 14 interviews were carried out by the lead researcher AP, who had no prior relationship with the participants and was not affiliated with the US course. The interviewees were told that AP had an interest in their views of learning US skills. The risk of respondent bias, in this case the tendency to give socially desirable answers, was minimised by careful question design. The interviewer was not much older than the participants, preventing a feeling of hierarchy. Only the interviewee and AP were present during the interviews, which lasted an average of 45 min and were audio-recorded. Short field notes were made after each interview, commenting on the mood of the interview and reflecting the interviewer’s role and potential influence. The interviews were conducted at a location of the participants’ choice, most often at a public venue or at the University. Interviews were conducted in Swiss German and transcribed verbatim using High German diction. The transcriptions were done by AP as well as BB and KL, two medical students, all of whom are native Swiss German speakers. We wrote and used a transcription manual to ensure that transcripts were consistent. No repeat interviews were carried out and interview transcripts were not returned to the participants. Analysis was conducted using the High German transcripts.

### Data analysis

AP, a medical doctor as well as a medical educator, did the primary analysis. She and MH, an experienced medical doctor, medical educator and qualitative researcher, independently coded three of the transcripts and compared their results to look for inconsistencies. Throughout the process, data was read and reread, using constant comparison with earlier data. Themes which derived from the data were labelled using a process of open coding to identify and later categorise phenomena in the data [24]. The interviews and analysis of the data were carried out in iterative cycles, going back and forth between the data gathering and analysis, allowing for new insights to inform changes to the interview guide [20, 25]. Both coders regularly revised and refined the codes. No software was used.

After 14 interviews, the question of data saturation was discussed among the research team (AP, MH, RH, BB). As no new themes were arising, it was agreed that data saturation had been achieved [26].

The research team reviewed the findings individually then met to discuss the thematic structure. Each member of the team first presented their initial thoughts as to the themes. The team identified and continuously reviewed the themes until there was consensus [21]. For the sake of clarity and focus, the analysis focused on ultrasound-related factors and excluded themes related to course design in general.

We sent an overview of the key themes to four of the participants and asked for their comments. No changes needed to be made as a result of this.

## Results

14 students participated in the interviews. Table 1 shows participants’ characteristics.

**Table 1:** Participants’ characteristics

Characteristic	Participants (*n* = 14)
Sex	
• Female	11
• Male	3
University	
• University of Bern	7
• University of Zurich	7
Previously completed another US course	2
Year of studies	
• Year 3	6
• Year 4	1
• Year 5	7
Age: median (range)	24 (22–25) years

We identified four US-specific themes that contribute to high levels of motivation to learn US skills. Table 2 shows the themes and subthemes.

**Table 2:** Themes and subthemes

Themes	Subthemes
1. **Perception of professional benefits**	- Benefits as a medical student - Benefits as a physician
2. **The exciting experience component**	- Fascination - Social aspects of learning US - A practical skill - Learning US as a challenge
3. **The positive nimbus of US**	- US as a modern tool - Advantages for patients
4. **The power of visualisation**	

The themes and subthemes are described in more detail below, with quotations identified by participant number.

### Perception of professional benefits

All participants expressed that learning US would be an advantage professionally. This could be during their studies, or as practicing physicians. Learning US as early as possible was seen as adding to this benefit.

#### Benefits as a medical student

Some participants wanted to understand US images shown in lectures: *Now, if we now have an US image in a lecture, I think “yes, ok, I can follow now”. (Participant(P)6)*

For most participants, revising and deepening their knowledge of anatomy, as well as applying that knowledge to the patient’s body, were key benefits and motivators. Many participants felt learning US improved their spatial thinking: *US gives you a different feeling of the relationships between the organs in the body. And uh, somehow you really know where the kidneys are or where the liver is. (P9)*

One participant appreciated that US also put into perspective what was clinically relevant for their future career: *And it was always reassuring for me that “yes, what is really relevant, I know” and if I no longer have the twenty-seventh branch of this artery in my head (…) so I was able to put in perspective the anatomical knowledge. For my future medical career. (P9)*

#### Benefits as a physician

All participants stated that having US skills could be relevant to their future as physicians. They thought it was important to understand the indications and be able to interpret US images. Many participants were certain US would be part of their clinical life. One participant stated: *Yes, it is also an examination method that is actually used in many disciplines. That means that sooner or later I will be asked to perform it on a patient. (P2)* Several participants felt that they needed to be well prepared for their transition from student to resident: *And that that’s my goal more or less, things that you can already acquire now, that I have them ready at hand. That at least that will work. (P2)* There was also the notion that having US skills would help *(…) avoid and reduce unnecessary imaging procedures and refer less patients to specialists. (P6)*

### The “exciting experience” component

For many participants, the motivation was connected to the feeling of experiencing something inherently fun and exciting: *So, yes, it’s also a fascinating, so it’s still a “wow”. A “wow moment”. (laughs) (P3).* Participants were intrinsically fascinated with the topic of US and the aspect of visualisation added to this: *What I really find interesting is that my fascination still doesn’t let up (…) well, I just found it fascinating to somehow see my own organs for once (laughs). (P9)*

#### The social aspects of learning US

Interpersonal factors played an important role, to learn and interact in small groups with other participants was described as similar to engaging in a new hobby, as well as a way of escaping everyday student life: *Um, and what did I always enjoy the most? I think it was simply the interaction with the others. It’s just something that we don’t have much of in our studies. (P9)*

While most participants said that the social aspect did not influence the decision to apply for the course, it was important for their positive perception of, and continuing engagement in, the course. They noted that the course gave them the opportunity to meet new people or do something with friends.

#### A practical skill

Many participants enjoyed doing something practical, as opposed to simply learning theory: *Yes, and that’s actually kind of cool. Yeah, not exactly like a surgeon, but still a little bit hands-on. Yes. (P3)*

They found learning US also enabled them to put their learning into a clinical perspective, giving them an insight into what they were working towards. One participant described how their US skills helped her become an active member of a clinical team during the practical year: *I was allowed to scan a child because I said I had done this course. And afterwards I felt like one of them, not just like a trainee standing at the back, but that I had been fully interactive. (P12)*

#### Learning US as a challenge

Participants found learning US to be a positive challenge: *If you just get it or, also with the kidney, if you get it and then you recognize everything, that’s a pleasure. (P11)*

Most participants performing US was not a natural gift but something they had to learn, requiring practice, diligence, and effort: *Sure, there’s a mega process behind it until it’s intuitive, and it’s not the case with me at all yet, but I can imagine that, that it’s, um, doable with practice. Like the driving test, maybe. (P13)*

### The positive nimbus of US

Many participants said that they believed US to be an up-and-coming modality in medicine, with many advantages. We summarise these recurrent themes under the concept of the “positive nimbus” of US, referring to its positive image in public and medical discourse.

#### US as a modern tool

Many participants stated that US was the diagnostic tool of the future, which would become part of every physician’s basic equipment. This was often something that they had heard from others (peers, lecturers, physicians): *(…) there was a doctor who said it was super important that you can do it and I should definitely take every opportunity. (P5)*

Participants had a positive impression of US as a tool, ascribing many favourable attributes to it, principally that it was fast and easy. The participants found US to be versatile in terms of when and where it could be used (which body part, but also which geographical location), and for which indications to use it. They commented that they were fascinated by the breadth of what can be done with US: *Yes, what I find interesting about US is that it can really be used in many different areas. (P7)*

#### Advantages for patients

US as a tool was also described as having advantages for patients because it did not entail radiation, was non-invasive, and not painful. Another benefit for patients was that they could watch the examination “live”: *You can show an image directly and that you can say to the patient “there it is and that’s how it is now. It’s good the way it is.” (P7)*.

### The power of visualisation

One key theme was the appeal of visualising things with US, and this contributed to the positive nimbus of US. For some participants, visualising organs was helpful because it matched their learning style: *Well, personally, I’m just very much a visual person. (P8)*

Most participants found it impressive to see inside the body and thereby gather a lot of information. It gave them a sense of security and the feeling that US was more objective and precise than other forms of examination: *So yes, it seems to me that US is a bit more objective. (P5)*

With the help of US, participants were easily able to confirm or exclude pathologies, which they found fascinating. Being able to capture an image was thought to be helpful too: some participants stated that it allowed them to show the image to others if they had any uncertainties, and to check that they had done it correctly: *Something that you can see can perhaps be better discussed with other people afterwards. (P8)*

## Discussion

### Principal findings

In this qualitative interview study, medical students gave a range of reasons for their high motivation to learn US: the positive image surrounding the modality in general, with an emphasis on the advantages of visualisation; they perceived potential professional benefits; and they described it as a fun, exciting activity and a way to interact positively with other learners.

### Interpretation of the results

The four themes we identified can be related to the three universal needs of autonomy, competence and relatedness described in SDT.

Mastering US skills contributed to a feeling of competence and autonomy. At the same time, feeling competent in performing US supported students in preparing for their transition from student to resident, giving them confidence in clinical situations and in interactions with patients, thus providing a professional advantage.

Learning US was described as an exciting and fun group activity. This perception aligns with the need for relatedness as described in SDT. The feeling that US was a practical alternation to their theoretical student life and a positive challenge map across to the need for competence.

The idea of learning to use a “modern”, technical tool, gave a feeling of autonomy and competence. Additionally, the fact that US is a mobile device compared to other imaging modalities was seen to offer a high degree of autonomy when deciding when and where to use it.

The visual aspect of US allows for just-in-time feedback and the immediate feeling of success when getting the right image, thus meeting the need for competence.

Whereas all four themes can be linked to the universal needs of SDT, the visual aspect of US and the positive nimbus surrounding US are more related to the “Zeitgeist”. In our study, participants’ comments suggested that they associated US with positive attributes and thought it would become increasingly important in the future. The sense that being able to visualise something was superior to other ways of examining– another subtheme we found– was central to this notion.

### Comparison with existing literature

Other studies confirm that students believe learning US is an advantage in their medical studies [11, 13, 14]. A cross-sectional survey of graduates confirms that early US training yields physicians who are better prepared to integrate it into clinical practice [27]. While the transition from medical school to residency differs depending on the national context, it has been described as stressful and challenging in studies from different countries [28, 29]. Early exposure to clinical environments in undergraduate years may help reduce the stress of this transition [29]. Integrating theoretical knowledge into practice in medical education has been an important issue in medical education for over a decade and remains so today. US teaching in undergraduate studies can aid professional identity formation [30]. Our results correspond with Wang et al.’s findings, that students are motivated to learn ultrasound because they believe it to be a necessity and/or advantage in their future work or current studies and because they want to learn out of interest. Wang et al. point out that relatedness played less of a role for students’ motivation, while in our study relatedness played an important role in the theme of “fun and exciting activity”. One possible explanation for this discrepancy could be that a distinction between motivation to initiate behaviour (e.g. sign up for a course) and to maintain motivation (e.g. stay motivated during the course) is seldom made. Participants in Wang et al’s study were questioned on motivation to initiate US learning, while in our study, participants had already begun or finished the US course and relatedness contributed to continuing motivation during the course but was seldom discussed as a reason for initial interest [19]. 

Our participants felt that visualisation enhanced the objectivity and reliability of an examination, providing a feeling of security, corresponding with Feilchenfeld et al.’s statement that “US’s visual evidence is viewed as truth, more accurate (truthful) than clinical information provided via other senses” [31]. This “Zeitgeist”, the “visuo-centric discourse” which claims the superiority of visual information over other data, may be common among medical students: a paper on body pedagogics states that “oculocentrism” (a perception of superiority of vision over other senses) is typical in Western society, especially in the medical profession [32]. The current discourse in our (medical) society that seeing is superior to, and more legitimate than, perceiving with other senses and is thus also beneficial for patient care seems to add to the “positive nimbus” of US and has an effect on students’ high motivation to learn the skill [26, 28].

### Strengths

This is the first qualitative study designed to study medical students’ motivation for US education. The design of the interview guide and the interpretation of the results were guided by a consistent theoretical framework (SDT). We interviewed participants from two different medical schools. We piloted the interview guides carefully, achieved data saturation and member-checked the findings.

### Limitations

Our participants were from a population taking part in a course with no curricular accreditation, making it likely that they had a high degree of motivation. The participants were students who volunteered to take part in the study, resulting in a risk of self-selection bias. We assumed that the students participating in a voluntary course and voluntarily taking part in the study would be especially highly motivated. Because our aim was to explore high levels of motivation for ultrasound, we hypothesise that the sample was in line with our research question. The factors motivating these students may not apply to other students, for example students with low motivation to learn ultrasound. Including a more varied sample might have given us other insights. Due to the COVID-19 pandemic, we could not stratify participants as intended, as US courses were put on hold for some time. Many students were busy with volunteer work at health facilities and may have been less willing to meet face-to-face. However, there was an equal number of participants from both Universities, and the gender distribution approximately reflects the gender distribution in Swiss medical studies. Also, the participants came from a homogenous group, which makes it more likely that the sample was adequate [33].

### Implications for research and practice

Understanding the mechanisms of motivation in medical education helps to create a supportive learning environment and to nurture autonomous motivation. While some of our findings were specific to the modality of US, some can also be applied to other teaching areas:

Visualisation aids learning, providing immediate feedback, a sense of competence and of autonomy, adding to the motivation. For areas where visualisation is not an option, educators should consider using alternative ways to give immediate feedback, when planning educational programmes. This could mean verbally providing immediate and constructive feedback, or using a portfolio to visually track learners’ progress.

Participants found learning US fun and exciting. Not all areas of learning allow for the hands-on experience of US education, but reflecting on where practical aspects can be integrated into teaching and how to challenge students optimally– making tasks not too easy nor too difficult– could enhance students’ motivation. One example is the “gameification” of learning content, which may add to the fun factor. Educators should enable group experiences and interaction when possible. This could be supported by encouraging students to actively participate in group work, giving a structure to educational units, while also providing enough room to learn in a self-directed way as a group. Facilitators to this can be a setting of mutual trust and open dialogue, and adjusting the learning environment, for example by considering seating arrangements.

Students are motivated to learn things that will benefit them professionally, making awareness of the clinical relevance of learning content essential. Explicitly addressing concerns about transitioning from medical school to residency and providing information on how a specific skill will help for this transition could increase students’ motivation to learn. Pointing out the relevance of the subject to medical practice could help less enthusiastic students see the importance of it and result in a more autonomous– rather than controlled– choice to learn something [34]. 

Educators should be aware that learning happens in the context and discourse of society, and that this can affect both educators’ and learners’ perspectives and motivation. While educators cannot influence the discourse directly, it is important to understand its existence, to modify barriers and facilitate motivation. The mechanisms of such discourses and their possible impact on students’ views require more research. 

Future quantitative studies could explore to which extent a more general medical student population relates to the identified themes in order to validate them. Including students with low motivation for ultrasound or students with high motivation in other areas as participants and comparing and contrasting these results with those of this study could also provide a more comprehensive view of motivational factors and be an interesting area for further research. Factors which initially sparked participants’ interest and motivation to sign up for the course may be different to those which maintained and increased their motivation during the course. Longitudinal studies would enable a deeper understanding of such nuances and other changes in motivation over time.

## Conclusions

We found four themes that have not yet been described in the literature: US was perceived as helpful in contextualising and exemplifying other learning content and supporting the transition into professional identity and, ultimately, clinical practice; the group setting with just-in-time feedback provides a fun and positive learning experience; participants reported being motivated to learn US because it was the tool of the future, and being able to visualise something trumped other forms of examination, reflecting the visuo-centric Zeitgeist. Knowledge and understanding of these themes may be relevant to other areas of medical education.

### Electronic supplementary material

Below is the link to the electronic supplementary material.

Supplementary Material 1

Supplementary Material 2

## Data Availability

The datasets used and analysed during the current study are available from the corresponding author on reasonable request.

## Abbreviations

US: ultrasound
P: participant
