# Supplementary material for: Why are medical students so motivated to learn ultrasound skills? A qualitative study
Source: BMC Med Educ. 2024 Apr 26;24:458. doi: 10.1186/s12909-024-05420-3 (PMC11046757; doi:10.1186/s12909-024-05420-3)
Supplement: Supplementary file 1 — Supplementary Material 1 [file 12909_2024_5420_MOESM1_ESM.pdf]

Appendix 1: Consolidated Criteria for Reporting Qualitative Research (COREQ): a 32-item checklist

No.	Item	Guide questions/descriptions	Notes
**Domain 1: Research Team and reflexivity**			
Personal characteristics			
1.	Interviewer/facilitator	Which author/s conducted the interview or focus group?	All 14 interviews were carried out by the lead researcher AP.
2.	Credentials	What were the researcher's credentials?	AP: MD, MMEd.<br>MH: MD, MB BS, FRCGP, MMEd.<br>RH: MD, MMEd.
3.	Occupation	What was their occupation at the time of the study?	AP was working at the hospital as a medical doctor as well as a medical educator at the Institute of Primary Healthcare, University of Bern at the time of the study.<br>MP was working as a primary care researcher and as a medical educator at the Institute of Primary Healthcare, University of Bern.<br>RH was working as a general practitioner and as head of education at the Institute of Primary Healthcare, University of Bern.
4.	Gender	Was the researcher male or female?	AP: female<br>RH, MH, BB: male
5.	Experience and training	What experience or training did the researcher have?	MH and RH are experienced qualitative researchers.<br>AP had prior experience in qualitative research.
Relationship with participants			
6.	Relationship established	Was a relationship established prior to study commencement?	AP had no prior relationship with the participants and was not affiliated with the US course.
7.	Participant knowledge of the interviewer	What did the participants know about the researcher?	The interviewees were told that AP had an interest in their views of learning ultrasound skills.
8.	Interviewer characteristics	What characteristics were reported about the interviewer/facilitator?	The research team had an interest in what contributes to students' motivation to learn ultrasound.
**Domain 2: study design**			
Theoretical framework			

9.	Methodological orientation and Theory	What methodological orientation was stated to underpin the study?	This was a qualitative study following a constructivist paradigm and using thematic analysis to analyse the data.
Participant selection			
10.	Sampling	How were participants selected?	Purposive sampling was used.
11.	Method of approach	Method of approach How were participants approached?	Potential participants were invited by an email forwarded to them by the course organisers and were also approached directly at US courses.
12.	Sample size	How many participants were in the study?	14 medical students were interviewed.
13.	Non-participation	How many people refused to participate or dropped out? Reasons?	After initial contact had been made, none of the participants declined to take part in the study or dropped out of it.
Setting			
14.	Setting of data collection	Where was the data collected?	The interviews were conducted at a location of the participants' choice, most often at a public venue or at the University.
15.	Presence of non-participants	Was anyone else present besides the participants and researchers?	Only the interviewee and AP were present during the interviews.
16.	Description of sample	What are the important characteristics of the sample?	The median age of the participants was 24 years. 11 of the 14 participants were female. Half of the participants attended the University of Bern, the other half the University of Zürich.
Data collection			
17.	Interview guide	Were questions, prompts, guides provided by the authors? Was it pilot tested?	The interview guide was semi-structured. It consisted of 10 main questions which focused on participants' reasons to learn US and the perceived benefits (see Appendix 2). Each main question had probes to get more in-depth information. The interview guide was piloted with two students, and changes were made where necessary.
18.	Repeat interviews	Were repeat interviews carried out? If yes, how many?	No repeat interviews were carried out.
19.	Audio/visual recording	Did the research use audio or visual recording to collect the data?	The interviews were audio-recorded.
20.	Field notes	Were field notes made during and/or after the	Short field notes were made after each interview, commenting on the mood of the interview and

		interview or focus group?	reflecting the interviewer's role and potential influence.
21.	Duration	What was the duration of the interviews or focus group?	Each interview lasted an average of 45 minutes.
22.	Data saturation	Was data saturation discussed?	After 14 interviews, the question of data saturation was discussed among the research team (AP, MH, RH, BB). As no new themes were arising, it was agreed that data saturation had been achieved.
23.	Transcripts returned	Were transcripts returned to participants for comment and/or correction?	Interview transcripts were not returned to the participants.
**Domain 3: analysis and findings**			
Data analysis			
24.	Number of data coders	How many data coders coded the data?	AP and MH independently coded three of the transcripts and compared their results to look for inconsistencies. AP coded the rest of the interviews with regular feedback of the research team.
25.	Description of the coding tree	Did authors provide a description of the coding tree?	A description of the coding tree was not provided.
26.	Derivation of themes	Were themes identified in advance or derived from the data?	Themes derived from the data in an inductive manner.
27.	Software	What software, if applicable, was used to manage the data?	No software was used.
28.	Participant checking	Did participants provide feedback on the findings?	We sent an overview of the key themes to four of the participants and asked for their comments. No changes needed to be made as a result of this.
Reporting			
29.	Quotations presented	Were participant quotations presented to illustrate the themes / findings? Was each quotation identified?	Key findings were illustrated with quotations. Each quotation was identified with a participant number.
30.	Data and findings consistent	Was there consistency between the data presented and the findings?	All findings were derived from the data and supported by illustrative quotes.
31.	Clarity of major themes	Were major themes clearly presented in the findings?	Major themes and subthemes are clearly identified in Table 2: Themes and subthemes, as well as in the headings.
32.	Clarity of minor themes	Is there a description of diverse cases or	(Sub-)themes that were only described by one

discussion of minor themes?	participant are also discussed in the results section.
