# Supplementary material for: Why are medical students so motivated to learn ultrasound skills? A qualitative study
Source: BMC Med Educ. 2024 Apr 26;24:458. doi: 10.1186/s12909-024-05420-3 (PMC11046757; doi:10.1186/s12909-024-05420-3)
Supplement: Supplementary file 2 — Supplementary Material 2 [file 12909_2024_5420_MOESM2_ESM.pdf]

Appendix 2: Interview guide “motivation to learn ultrasound” (translated from German into English)

<u>Beginning:</u>	
• What did you know about ultrasound before you started your ultrasound training?<br>• How far along are you with your ultrasound training?	→ Which ultrasound courses did you take / what experience did you have in ultrasound before you started your ultrasound training?
<u>Reasons to learn ultrasound:</u>	
• How did you come to attend the ultrasound course?	→ What made you decide to enrol? / what interested you in learning ultrasound? → Why?<br>→ Were there any other things that aroused your interest in the course? → Which ones?<br>→ Are there role models for you, for example?
• Can you remember the first time you heard about ultrasound lessons? What was your first thought?	→ To what extent has this impression changed?
• Tell me about your first ultrasound, which you performed yourself.	→ How did that go?<br>→ What did that feel like for you?<br>→ How is that feeling different today?
• Tell me about a typical ultrasound lesson. What happens?	→ What is especially fun for you?<br>→ What is particulary difficult for you?<br>→ Is there anything you don’t like so much? What?<br>→ How do you experience the atmosphere in the group?<br>→ How do you experience the physical closeness you have when practising?<br>→ What role does the social aspect of learning ultrasound play for you?<br>→ How does it feel for you to operate the device?<br>→ For some students, it also has something to do with feeling like a "real doctor". What are your thoughts on that?

• You also learnt other practical clinical skills, e.g. cardiac auscultation or venipuncture. How do those experiences differ from learning ultrasound?	→ How easy do you think it is to learn ultrasound compared to other diagnostic skills? → To what extent does this play a role for you?<br>→ Which role does visual control by the teacher play?<br>→ Which role does it play for you that you can see something and not just feel or hear it?<br>Several senses at the same time (feel, see, hear)
<u>Benefits of learning ultrasound / attending the course:</u><br><br>• Which advantages do you see in learning ultrasound skills?	→ Does this help with other aspects of your studies?<br>→ For example, some students find it helps with their studies, with learning anatomy. What do you think?<br>→ With ultrasound, you can look into the body yourself, "live". Could that also play a role?<br>→ Some find it helpful to better understand other imaging modes. How do you see this?<br>→ Diverse, practical, useful<br>→ To what extent do you think these skills will play a role in your later professional life?<br>→ What do you hope to achieve with the ultrasound skills you have learnt?<br>→ Can be used in very different contexts, not limited.
• This course consists of e-learning modules accompanied by practical courses. How important is the teaching format of the ultrasound course for you?	→ What are the advantages of this form of teaching for you?<br>→ What is the atmosphere like on the ultrasound course?<br>→ And the tutors?<br>→ How is it for you that you are very independent in where and when you learn?

	→ Where do you normally study?
• Would you recommend the ultrasound course to a friend?	→ Why? Why not?<br><br><u>Other aspects:</u><br>- Does it play a role for you, that you’re operating a technical device? In which way?<br>- Challenge, curiosity<br>- How enthusiastic are you about the course? Why?<br>- How competent do you feel doing ultrasound?
<u>Closing:</u><br><br>• Is there anything else you would like to say on this topic that we haven't discussed yet?	<u>General in-depth probes:</u><br>→ Can you explain this in more detail?<br>→ Do you have an example of this?<br>→ What do you mean exactly?<br>→ That's interesting, tell me more.
